# Artificial Neural Network Analysis in Preclinical Breast Cancer

**Published:** 2013-11-20

**Authors:** Gholamreza Motalleb

**Affiliations:** Department of Biology, Faculty of Science, University of Zabol, Zabol, Iran

**Keywords:** Neural Network Model, Breast Cancer, Virotherapy

## Abstract

**Objective:**

In this study, artificial neural network (ANN) analysis of virotherapy in preclinical breast cancer was investigated.

**Materials and Methods:**

In this research article, a multilayer feed-forward neural network trained with an error back-propagation algorithm was incorporated in order to develop a predictive model. The input parameters of the model were virus dose, week and tamoxifen citrate, while tumor weight was included in the output parameter. Two different training algorithms, namely quick propagation (QP) and Levenberg-Marquardt (LM), were used to train ANN.

**Results:**

The results showed that the LM algorithm, with 3-9-1 arrangement is more efficient compared to QP. Using LM algorithm, the coefficient of determination (R^2^) between the actual and predicted values was determined as 0.897118 for all data.

**Conclusion:**

It can be concluded that this ANN model may provide good ability to predict the biometry information of tumor in preclinical breast cancer virotherapy. The results showed that the LM algorithm employed by Neural Power software gave the better performance compared with the QP and virus dose, and it is more important factor compared to tamoxifen and time (week).

## Introduction

There is a need for new prognostic systems in cancer that can integrate an expanding number of prognostic factors (1). In order to generate the survival estimates of a patient, an optimized method is applied to evaluate both tumor specification and patient’s prognostic information (2). The use of artificial intelligence (AI) has become widely accepted in medical applications (3). Advantages of neural network methods are as follows: ease of optimization, cost-effective, flexible non-linear modeling of large data sets, accuracy for predictive inference, and with potential to support clinical decision making. These models can make knowledge dissemination easier by providing explanation, for instance using of extraction rule or sensitivity analysis (4). In patients with breast cancer, earlier studies have reported promising results for neural network models (5). Intra tumoral injection is the method *in vitro* therapy in order to delivery local viral genes in tumor tissues to decrease systemic toxicity (6). Avian paramyxo virus type1 (Newcastle disease virus) has been shown to have properties as an excellent anticancer agent (6). NDVAF2240 has been tested as an anticancer agent in vivo (7,8).

An interesting question is whether artificial neural network could improve the accuracy of predictions in order to obtain prognostic information of tumor during Intra tumoral injection of NDVAF2240 in breast cancer induced in Balb/c mice.

## Materials and Methods

**Fig 1:**
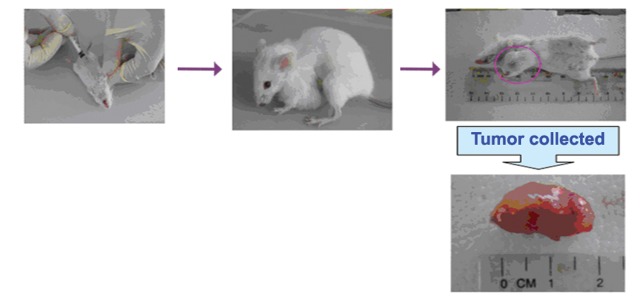
The representative pictures of mice with tumor before and after sacrificing.

In this research study, tumor development was evaluated according to modified method of Xanthopoulos as carried out previously (7). Briefly, 200 females Balb/c mice were divided randomly into 10 cancerous groups consisting of 20 mice per group. The mice were initially induced with 10^4^ 4T1 cells, NDV-AF2240 and tamoxifen co-culture. Cancerous groups were divided into cancer control (CC); cancer treated with 0.5 μg/ml tamoxifen citrate (CT); cancer treated with 8, 16, 32 and 64HA units of NDV-AF2240 named as C/NDV8, C/ NDV16, C/NDV32, and C/NDV64,respectively; as well as cancer treated with 8, 16, 32 and 64HA units of NDV-AF2240 and tamoxifen named as CT/ NDV8, CT/NDV16, CT/NDV32 and CT/NDV64, respectively, daily for four weeks. The tumor was detected by palpation around the induction area. Tumor size, volume and weight were measured weekly as described before (7). The collection of tumor was done weekly. Five mice from each group were sacrificed with diethyl ether (Fig 1). All procedures were approved by international guidelines and by the Institute Research Ethics and Animal Care and Use Committee of (University Putra Malaysia. Every effort was made to minimize the number of animals used and their suffering.)

### Statistical methods

A commercial artificial neural network (ANN) software, known as Neural Power version 2.5 (CPC-X Software, USA) was applied throughout this study. The software has been also used by other researchers (9-15). This software is a Windows®- based package supporting several types of training algorithms. Neural Power operates via a graphical user interface (GUI) and enables a user to load the training and test sets, design the network architecture, select the training algorithm and generate the individual models for each output variable in a single operation (15).

### Data sets

In order to determine the optimum number of neurons in hidden layer, a series of topologies was examined, in which the number of neurons was varied from 1 to 20. The root mean square error (RMSE) was used as the error function. Decision on the optimum topology was based on the minimum error of testing. Each topology was repeated five times to avoid random correlation due to the random initialization of the weights (16). The experimental data used for ANN design are presented in table 1. The experimental data were randomly divided into the following three sets using the option available in the software: 24, 6 and 6 of data sets as training, testing and validation, respectively. The training data was used to compute the network parameters. The testing data was used to ensure robustness of the network parameters. To avoid the "over fitting" phenomenon, the testing stage was also used to control error; when it increased, the training was stopped (17). The validation data (or unseen data) was excluded from training, and testing was used to assess the predictive ability of the generated model (18).

### ANN description

**Fig 2:**
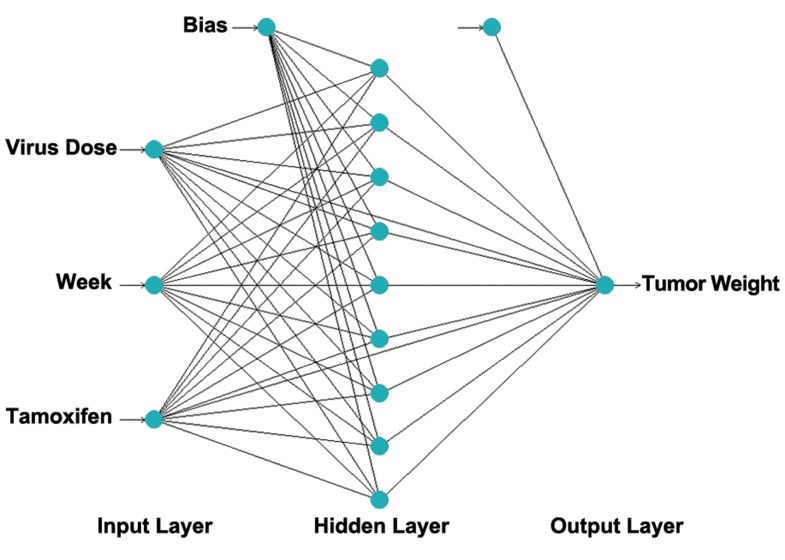
A multilayer feed-forward perceptron (MLP) network consisting of three inputs, one hidden layer with nine neurons and one output.

A multi-layer perceptron (MLP), based on feedforward ANN which uses back- propagation learning algorithm, was applied for modeling of breast cancer virotherapy. The network consists of an input layer with three neurons, a hidden layer with nine neurons and an output layer. Inputs for the network are virus dose, tamoxifen and week (time), while the output is tumor weight. The structure of proposed ANN is shown in figure 2.

**Table 1:** Experimental values, actual and model predicating tumor weight on the breast cancer virotherapy


Genes	Virus dose	Week	Tamoxifen	Tumor weight	
Training				Actual	Predicted

**1**	32	1	0	0	0.000008
**2**	64	1	0	0	0.000001
**3**	8	2	0	0	0.000649
**4**	16	2	0	0	0.000008
**5**	8	3	0	0	0.016458
**6**	16	3	0	0	0.000054
**7**	32	3	0	0	0.000000
**8**	64	3	0	0	0.000000
**9**	16	4	0	0	0.007790
**10**	64	4	0	0	0.000000
**11**	8	1	5	0	0.002089
**12**	16	1	5	0	0.012448
**13**	64	1	5	0.9825	0.984800
**14**	8	2	5	0	0.000378
**15**	32	2	5	1.62	1.618200
**16**	64	2	5	2.045	2.046900
**17**	16	3	5	0	0.000842
**18**	32	3	5	0.995	0.993780
**19**	64	3	5	2.27	2.258800
**20**	16	4	5	0	0.000259
**21**	64	4	5	1.8375	1.839400
**22**	0	1	0	0.6625	0.663030
**23**	0	2	0	0.335	0.334240
**24**	0	3	0	2.375	2.373500
**Testing**					
**25**	8	1	0	0	0.004600
**26**	64	2	0	0	0.000000
**27**	32	4	0	0	0.000006
**28**	16	2	5	0	0.002703
**29**	8	4	5	0	0.000082
**30**	0	4	0	2.635	2.633500
**Validation**					
**31**	16	1	0	0	0.000012
**32**	32	2	0	0	0.000000
**33**	8	4	0	0	0.091257
**34**	32	1	5	0.4225	0.269530
**35**	8	3	5	0	0.000485
**36**	32	4	5	1.895	0.273280
					


Scaled data are passed into the input layer, and then is propagated from input layer to hidden layer, and finally to the output layer of the network (19). In output layer, each node links and changes the inputs of previous layer as a junction summation using the following formula (20):

(1) $$y_{i}=\sum_{J=1}^{i}X_{i}w_{j}+b_{j}$$

where is the net input to node in hidden or output layer, is the inputs to node (or outputs of previous layer), wij is the weight representing the strength of the connection between the ith node and jth node, is the number of nodes and is the bias associated with node. Each neuron consists of a transfer function expressing internal activation level. Output from a neuron is determined by transforming its input using a suitable transfer function (21). Generally, the transfer functions for function approximation (regression) are sigmoidal function, hyperbolic tangent and linear function (20). The most popular transfer function for non-linear relationship is the sigmoid function (15,22-24). The general form of this function is as follows (20):

(2) $$z_{j}=\frac{1}{1+e^{{\mathrm{-y}}_{y}}}$$

z_j_, the output of node , is also an element of the inputs to the nodes in the next layer. In this study, the sigmoid function was used as the transfer function for the hidden and output layer nodes. The algorithms used to train ANN in this study are quick propagation (QP) and Leven berg- Marquardt back propagation (LM). The details of the algorithms have been reported elsewhere (15).

### Model evaluation

The performance of the ANN models is assessed on the basis of the root mean squared error (RMSE) and the coefficient of determination (R^2^) between the predicted values of the network and the actual values, which are calculated as follows:

(3) $$\mathrm{RMSE}={\left(\frac{1}{n}\sum_{i=1}^{n}{\left(y_{i}-y_{a}\right)}^{2}\right)}^{\frac{1}{2}}$$

(4) $$R^{2}=1=\frac{\sum_{i=1}^{n}{\left(y_{i}-y_{a}\right)}^{2}}{\sum_{i=1}^{n}{\left(y_{a}-y_{m}\right)}^{2}}$$

is the number of points, yi is the predicted value obtained from the neural network model, ydi is the actual value, and ym is the average of the actual values. The R^2^ shows the level of model fitness (25). If value of R^2^ is closer to 1, the model is considered as a better design and fits to the actual data (26). So, we considered the ANN model with lowest RMSE and highest R^2^ as the best ANN design (27-29).

## Results

**Fig 3:**
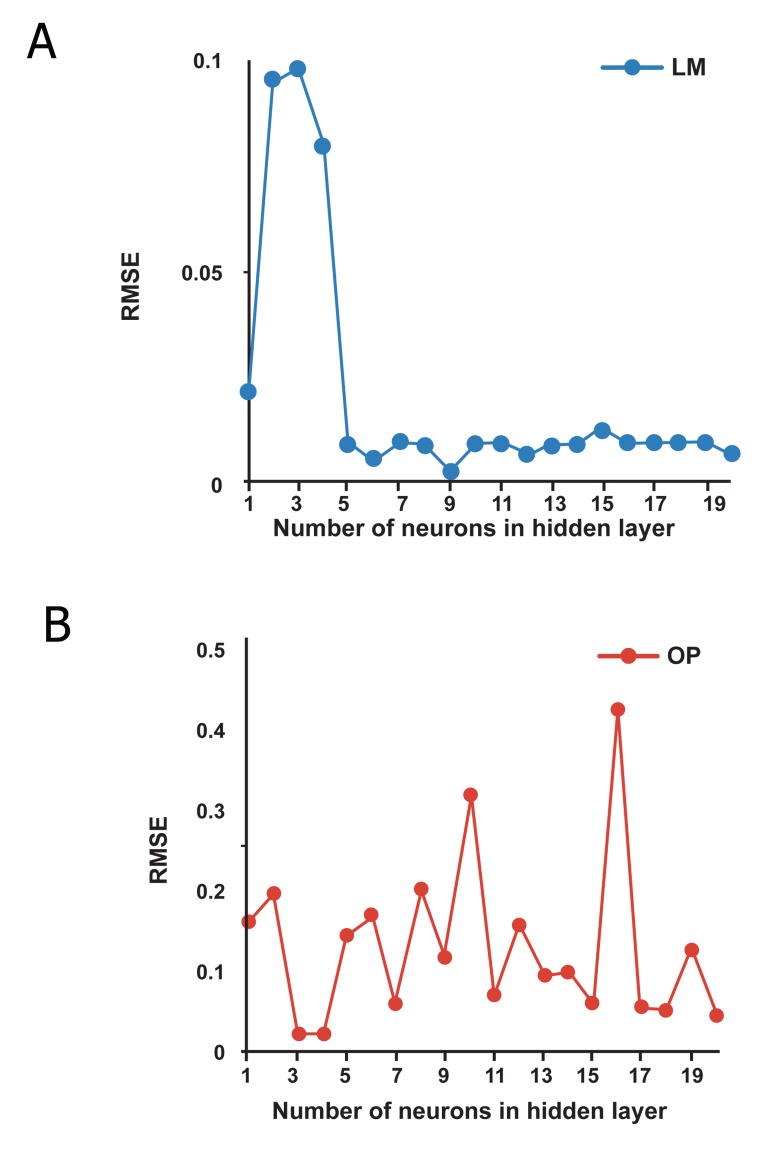
The performance of the network at different hidden neurons using: A. LM algorithm and B. QP algorithm.

The ANN was employed to create and predict a model in order to determine which factors, including virus dose, week (time) and tamoxifen, is the most important one during our preclinical *in vivo* study. Figure 2 illustrates the performance of the network for testing data versus the number of neurons in the hidden layer using LM and QP algorithms. After repeated trials, it was found that a network with 9 hidden neurons produced the best performance when LM algorithm was employed. However, a network with 3 hidden neurons produced the best result for QP algorithm (Fig 3). These topologies have lowest RMSE for the testing sets.

**Fig 4:**
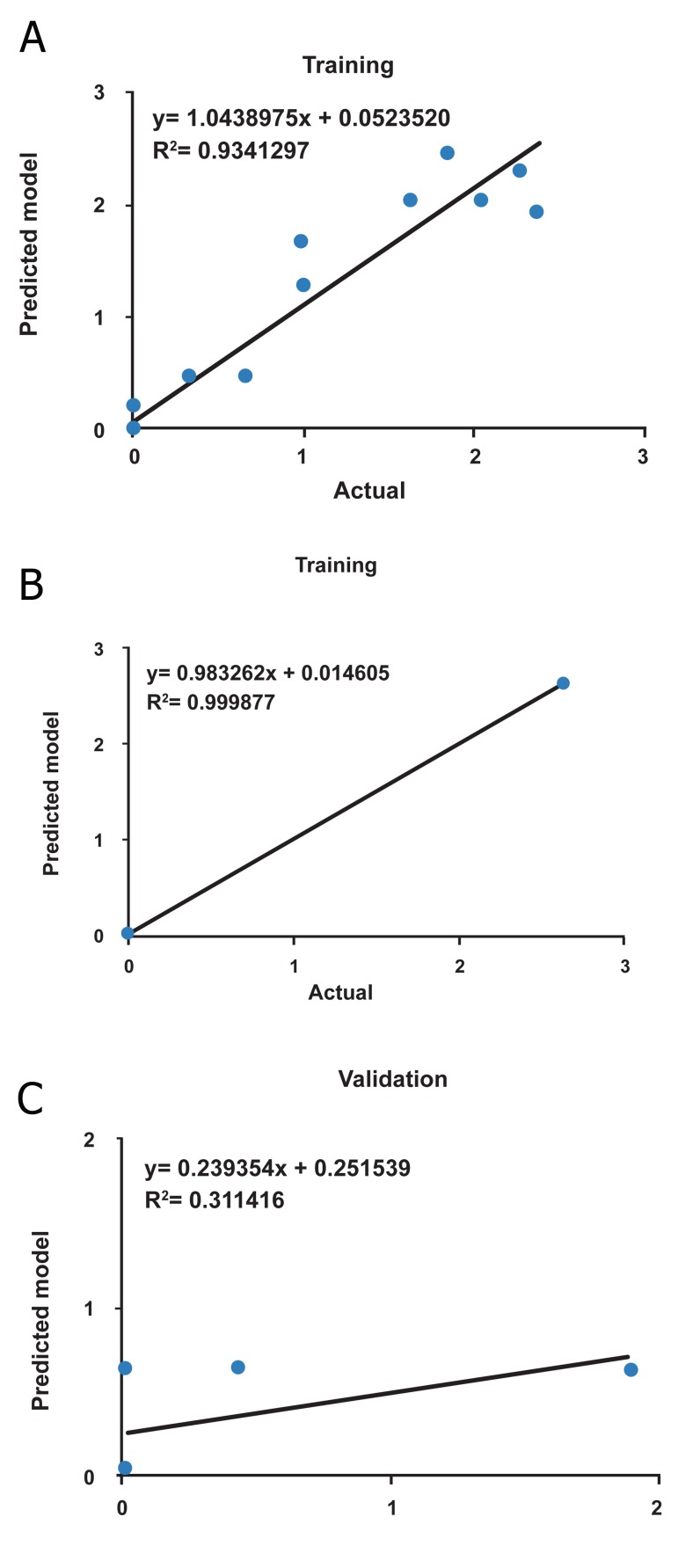
The scatter plots of ANN predicted tumor weight versus actual tumor weight for training (A), testing (B) and validation(C) data set using QP algorithm.

**Fig 5:**
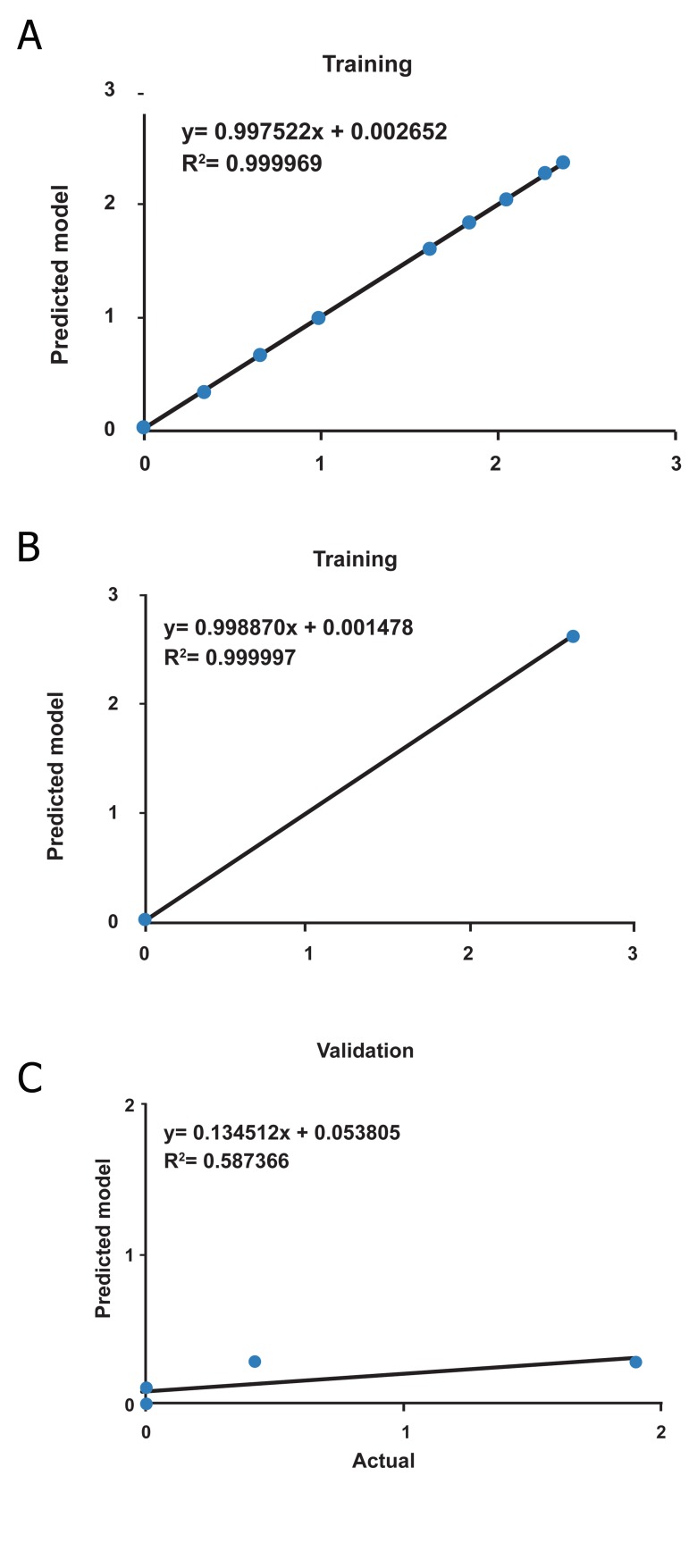
The scatter plots of ANN predicted tumor weight versus actual tumor weight for training (A), testing (B) and validation(C) data set using LM algorithm.

**Fig 6:**
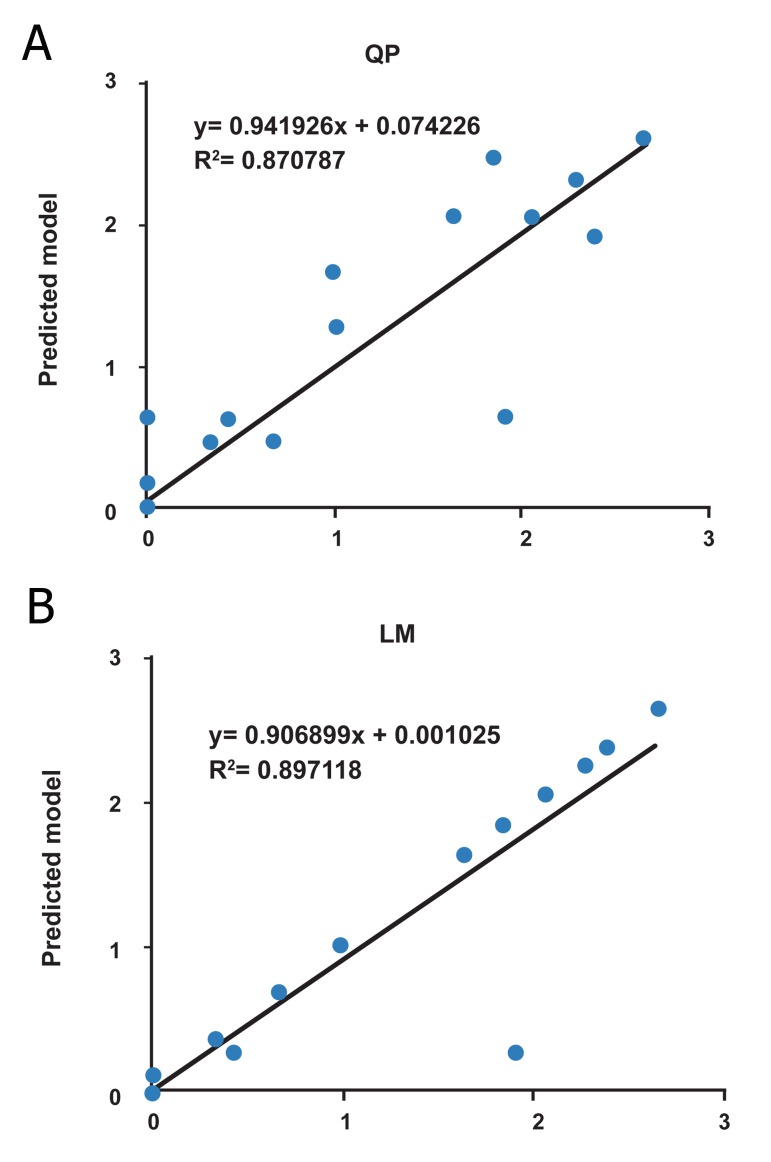
The scatter plots of ANN predicted tumor weight versus actual tumor weight for all data set using QP algorithm (A) and LM algorithm (B)

**Fig 7:**
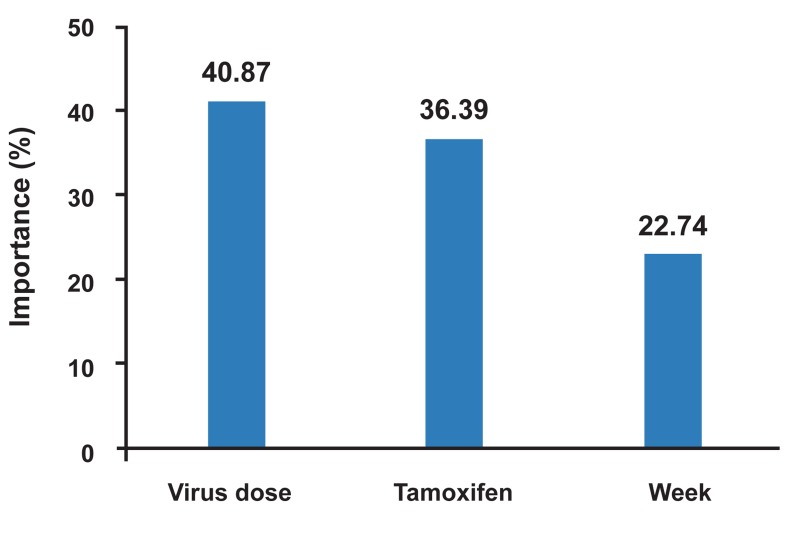
The importance of independent variables in the constructed ANN model.

The R^2^ and RMSE for two algorithms are presented in table 2. The LM algorithm has a better performance compared to QP algorithm (for all data, RMSE=0.271946 and R^2^ =0.897118, Table 2). Figures 4 and 5 show the scatter plots of ANN predicted value versus actual value with QP and LM algorithms for the training, testing and validation sets, respectively. The scatter plots for all data using QP and LM algorithms are shown in figure 6. Therefore, it could be concluded that model trained with LM algorithm is more efficient compared to QP model. In figure 7, the importance of selected variables in the construction of the ANN model using LM algorithm is shown. Interestingly, the virus dose showed higher contribution than the tamaxifen and time (week).

**Table 2:** The RMSE and coefficient of determinations (R^2^) are two learning algorithms on modeling in preclinical breast cancer


		Training		Training		Validation		All data	
Learning algorithm	The best architecture	RMSE	R^2^	RMSE	R^2^	RMSE	R^2^	RMSE	R^2^

**Quick propagation (QP)**	3-3-1	0.243624	0.934129	0.020922	0.999877	0.582782	0.311416	0.310242	0.870787
**Levenberg-marquardt (LM)**	3-9-1	0.005157	0.999969	0.002261	0.999997	0.666038	0.587366	0.271946	0.897118


## Discussion

At the present study, ANN analysis of Newcastle disease virus in preclinical breast cancer was investigated. The RMSE was used as the error function. The R^2^ was used as a predictive ability of the network. The aim of a cancer monitoring program is to detect tumors at early stage in order to have a successful treatment. A screening tool should not be expensive and invasive in order to permit its widespread application (30). Toxicity is very important in any experimental therapeutic agent, and oncolytic viruses are not different in killing cancer cells. Also, toxicity is dependent on the virus strain, genetic changes, and the dose of virus (31). Tamoxifen is used in treatment of steroid receptor positive breast cancer as a standard endocrine therapy (32). An artificial neural network model contains hundreds of artificial neurons combined through weights, which is also described as coefficients, are adjustable factors, so neural network (NN) is considered as a system with parameters. The weighed sum of the inputs constitutes the activation of the neuron. The activation signal is passed through transfer function to produce a single output of the neuron. Coefficients optimization in training continues until prediction errors is minimized, and the system gets accuracy with specified level. New input data or information can be given to the network when it is trained and tested. At this step, optimized coefficients or weights ratio shows the incorporation percentage in final result or output for each input data or parameter that could be computed as an important value (33). I selected dose of virus, week and tamoxifen as the main input layer factors. The findings of my ANN model is in agreement with study of Motalleb et al. (7), in which we showed changes of tumor weight and mass were dose-response during intratumoral injection of virus (Fig 7). From another point of view, my ANN model compared factors of virus dose, tamoxifen and time, among which the dose of virus is more important factor. Direct administration and intratumoral injection of different strain of NDV in clinical trials have been applied to cancer patients and the results showed this subject is required further evaluation. It is noted that it has been shown that intratumoral injection of NDV induce the tumor regressions significantly (34). For all data, the R^2^ of LM was 0.897118 that maybe due to the multiple factors involving in immunity situation of mice during the *in vivo* study.

To say in a different way, in biomedical research, the behavior of data is not fully predicted, and for this reason, the R^2^ for all data was less than R^2^ for testing. The research studies have been confirmed the dominant role of apoptosis by NDV-induced cell death in cancer research. NDV induces apoptosis through the following steps: viral entry into the cell, its replication, synthesis of its protein, and activation of caspases. NDV promotes oncolytic activity in tumors by different mechanisms including multinucleated formation of syncytia, activation of intrinsic and extrinsic apoptotic pathways, endoplasmic reticulum (ER) stress pathway activation, mitogen activated protein kinases (MAPK) pathways and secretion of pro inflammatory cytokines and chemokines (34). The model that was gained at this research could be very useful in saving time, cost and energy in pharmacology and viro-therapy of cancer research before going to clinical trial phase in human. However, we have to find new methods in clinical application to drop the disadvantages of intra tumoral injection of virus in gene therapy. To say in a different way, ANN can improve the accuracy of cancer survival prediction.

## Conclusion

In this study, the ANN predictions in order to obtain prognostic information of tumor during Intra tumoral injection of NDV-AF2240 in preclinical breast cancer have been optimized through a proper selection of the training algorithm. Different ANNs, trained with QP and LM, were evaluated with respect to their predictive ability. The LM algorithm employed by Neural Power software showed the better performance compared with QP. The results showed virus dose is more important factor compared to tamoxifen and time (week). It can be concluded that the ANN model of this research paper has good ability to predict the biometry information of tumor in preclinical breast cancer virotherapy.
